# Dynamics of Serological and Mucosal Antibody Responses against African Swine Fever Viruses in Experimentally Infected Pigs

**DOI:** 10.1155/2023/9959847

**Published:** 2023-02-25

**Authors:** Qingyun Xie, Yun Bai, Wan Wang, Rong Chen, Huixuan Xing, Yuzi Wu, Guoqing Shao, Zhigao Bu, Dongming Zhao, Zhixin Feng

**Affiliations:** ^1^Institute of Veterinary Medicine, Jiangsu Academy of Agricultural Sciences, Key Laboratory of Veterinary Biological Engineering and Technology, Ministry of Agriculture, Nanjing 210014, China; ^2^State Key Laboratory of Veterinary Biotechnology, Harbin Veterinary Research Institute, Chinese Academy of Agricultural Sciences, Harbin 150069, China; ^3^Institute of Animal Science, Tibet Agricultural and Animal Husbandry College, Tibet 860000, China

## Abstract

African swine fever virus (ASFV) is a lethal swine pathogen, and there is no effective vaccine or treatment available for ASFV infection. Recently, the occurrence of ASFV genotype I and genotype II natural mutants that manifest as subacute, longer-incubation, or persistent infections poses threats to preventing ASFV infection. The dynamics of antibody responses to ASFV are still completely unrevealed, especially the secretion of mucosal antibodies in oral fluid. Here, a systematic analysis was performed of serological and mucosal antibody secretion against 6 ASFV antigens after direct or indirect infection with four different ASFV strains or genotypes, namely, the field virulent genotype II isolate ASFV HLJ/18, the artificially attenuated genotype II strain HLJ/18-7GD, the naturally attenuated genotype II isolate HLJ/HRB1/20, and genotype I isolate SD/DY-I/21. Severe clinical signs of HLJ/18 infection were observed in pigs from 4 days postinoculation. However, no clinical signs were observed in HLJ/18-7GD-infected pigs. The contact pigs cohoused with the pigs intramuscularly infected with the isolate SD/DY-I/21 or HLJ/HRB1/20 only showed chronic clinical signs. Interestingly, the oral fluid sIgA responses to all the selected antigens were significantly stronger and earlier than the serum IgG responses in both HLJ/18- and HLJ/18-7GD-challenged pigs. Although significant fluctuations and individual differences appeared in oral swab sIgA responses in the contact transmission group, they were earlier than the corresponding serological IgG responses. Moreover, according to the comparative analysis of the three infection groups, P54 was proposed to be a dominant target for serological IgG diagnosis, while P30, CD2v, P54, P22, and P10 were more advantageous as mucosal sIgA diagnosis targets. These results highlight the important role of mucosal antibodies in the early diagnosis of ASFV infection and can provide references to screen appropriate targets for ASFV detection.

## 1. Introduction

African swine fever (ASF), caused by the African swine fever virus (ASFV), is a devastating haemorrhagic infectious disease in wild boar and domestic pigs, with acute infections associated with 100% mortality [1, 2]. Since the first report of ASF in Kenya in 1921, the disease has been sequentially identified in animals in Western Europe, South America, and Eastern Europe [3, 4]. The virulent genotype II ASFV was first detected in China in August 2018 and rapidly spread to 10 countries in Asia within a year [5–7]. Subsequently, naturally occurring lower-virulent genotype II variants and low-virulent genotype I viruses were detected in the field in China [8, 9]. The emergence of ASFV in Asia poses serious threats to global swine farming, leading to enormous socioeconomic losses.

Currently, the detection of lower-virulent mutants associated with nonlethal, subacute, long-incubation, or persistent infections has caused great difficulty in the precise prevention and control of ASF [8–10]. Due to the low level of virus loading, intermittent shedding, and larger individual differences in animals infected with these lower-virulent genotype II mutant strains and genotype I viruses [10], qPCR detection of ASFV is limited. At present, synchronous detection of antigens and antibodies has been attempted in clinical practice to improve detection accuracy, but ASFV antibody detection technology needs to be greatly improved, especially regarding sensitivity. Additionally, since no treatment or effective commercial vaccine against ASFV infection is available [11–13], an immunological test for early monitoring is desirable [14–16]. However, understanding of the secretion kinetics of antibodies against ASFV and knowledge of serological responses to virulent strains versus low-virulent strains are limited, hindering the selection of promising candidate antigens.

ASFV commonly enters the host via the tonsils or dorsal pharyngeal mucosa and then invades mandibular or retropharyngeal lymph nodes, subsequently spreading to the whole organism via the blood and activating systemic immunity, causing high fever, anorexia, depression, polypnea, skin redness, congestion, and cyanosis in swine [17, 18]. The protective mucosae comprising the oral/nasal epithelium and its associated lamina propria are considered the host's first line of defence against ASFV, inducing a mucosal immune response. Local mucosal immunity and systemic immunity constitute the defence system against ASFV. Previous studies on antibody secretion have shown that the sIgA response, the main effector of mucosal immunity, occurs significantly earlier than the systemic humoral immune response (IgG) to mycoplasma [19], influenza A virus [20], and Zika [21] virus infection, indicating that mucosal immunity may play a crucial role in the early recognition and minimalization of viral spread. Indeed, based on the cross-sectional and longitudinal monitoring of ASFV prevalence in local pigs in Kenya and Uganda, the researchers found that the isotype of the antibodies from these animals were strongly IgA biased relative to control domestic pigs and warthogs, suggesting that it has a role in mucosal immunity [22]. In addition, previous studies on the immune response to the naturally non-haemadsorbing isolate ASFV/NH/P68 showed marked hypergammaglobulinemia involving plasma IgG1, IgG2, IgM, and IgA isotypes [23].

Here, P30, P54, P72, P10, P22, and CD2v of ASFV, which were previously demonstrated to be highly expressed at different ASFV infection stages and to induce neutralizing antibodies against ASFV [24], were selected as target antigens in the dynamics analysis of serological and mucosal antibody responses. P30 and p54 are involved in virus invasion and are early expression proteins of ASFV [25]. In addition, P54 is required for viral membrane protein formation [26]. P72 is also involved in virus invasion and is the main nucleocapsid protein of ASFV, which is highly expressed in the late stage of infection. P10 is a principal serological epitope screened from antibody screening of a cDNA library using serum from a pig that had survived infection with ASFV, as well as P54 and P72 [27]. CD2v is a glycoprotein that binds red blood cells to infected cells and extracellular virions, and is embedded in the outer surface of the viral envelope [28]. Using the antigens selected above and four ASFV strains with different virulence including the field virulent genotype II isolate ASFV HLJ/18, the artificially attenuated genotype II strain HLJ/18-7GD [29], the naturally attenuated genotype II isolate HLJ/HRB1/20 [8], and genotype I isolate SD/DY-I/21 [9], this study aims to compare the differences in the secretion pattern of mucosal antibodies and serum antibodies, the differences in the kinetics of antibody responses to virulent and attenuated ASFV strains, and the differences in the antibody responses to different ASFV antigens. Understanding the correlation between antibody secretion and disease progression will support the development of early diagnosis and further study of the immunopathogenesis of ASFV-specific host immune responses.

## 2. Material and Methods

### 2.1. Antigen Production

The proteins p72, p10, p22, p54, p30, and CD2v of ASFV were expressed in an *E. coli* or baculovirus expression system, purified using Ni NTA affinity columns (GenScript Laboratories), and identified by SDS-polyacrylamide gel electrophoresis (Supplementary material (Available here)). The purified proteins were dialyzed in PBS and concentrated in ultrafiltration tubes (Millipore), and the protein concentrations were determined with a BCA protein assay kit (Beyotime Laboratories).

### 2.2. Animal Experiments

All animal assays were performed at the Laboratory Animal Center of the Harbin Veterinary Research Institute (HVRI). All ASF viruses were cultured in HVRI, and the TCID50 method was used for titer determination. All experiments with live ASF viruses were conducted within the enhanced biosafety level 3 facilities in the HVRI. SPF Large White and Landrace-crossed pigs aged 50 days obtained from the Laboratory Animal Center of the HVRI were used. Before being challenged with ASFVs, the pigs were confirmed to be free of antibodies against ASFV, classical swine fever (CSF), porcine respiratory and reproductive syndrome (PRRS), and pseudorabies virus (PRV) using nucleic acid detection and serum antibody ELISA detection in parallel. For the ASFV virulent strain-challenged group, four pigs were housed in an isolation room and were orally and nasally challenged with 10^5^ HAD_50_ of Pig/Heilongjiang/2018 (HLJ/18), a virulent genotype II strain [7, 30]. For the ASFV attenuated strain-challenged group, four pigs were housed in an isolation room and were orally and nasally challenged with 10^6^ TCID_50_ of HLJ/18-7GD, an artificially attenuated genotype II strain [29]. For the ASFV contact transmission group, two pigs were cohoused with four infected pigs that were intramuscularly inoculated with 10^3^ TCID_50_ and 10^6^ TCID_50_ of genotype I ASFV Pig/Shandong/DY-I/2021 (SD/DY-I/21) [8] or 10^6^ TCID_50_ of genotype II ASFV Pig/Heilongjiang/HRB1/20 (HLJ/HRB1/20) [9] from the first day of challenge.

### 2.3. Sample Collection

All samples were provided by the Laboratory Animal Center of HVRI and were processed as described previously [29]. Serum samples and oral fluid samples from HLJ/18-infected pigs were collected once every two days and once a day until 8 days postinoculation (DPI), respectively. Serum samples and oral fluid samples from HLJ/18-7GD-inoculated pigs were collected every five days until 60 DPI. Serum samples and oral swab samples from contact transmission pigs were collected on alternate days until 28 DPI. The pen-based oral fluid samples were collected from a pressed rope with knots hung in a pigsty that could be chewed by the animals. After soaking in sterile PBS overnight at 4°C, the supernatants were obtained by centrifugation at 10000 × *g*for 20 min at 4°C. Then, oral fluid samples were aliquoted into tubes and stored at −70°C. After being left at 4°C overnight, the serum samples were centrifuged at 3000 × *g*for 20 min. Then the supernatants were collected and stored at −70°C.

### 2.4. Nucleic Acid Detection of ASFV

The quantity of the ASFV genome in the samples was determined using quantitative real-time polymerase chain reaction (qPCR) published by the international epizootic office (Section 2.8, OIE Terrestrial Manual, 2012). The DNA was extracted according to the genomic DNA extraction kit (AxyGen, USA), and then amplification of the ASFV genome target B646L (P72) gene was executed using the following primers: 5′-CTG CTC ATG GTA TCA ATC TTA TCG A-3′, 5′-GAT ACC ACA AGA TC(AG) GCC GT-3′, and 5′-Fam-CCA CGG GAG GAA TAC CAA CCC AGT G-Tamra-3′ for qPCR. The quantity of ASFV genome was calculated using the standard curve after the procedure included 1 cycle at 50°C for 2 min, 1 cycle at 95°C for 10 min, and 40 cycles consisting of 15 s denaturation at 95°C and 1 min annealing at 58°C.

### 2.5. Serum IgG Responses to ASFV Antigens in Challenged Pigs

To detect serum IgG against ASFV, IgG-specific indirect ELISAs based on different antigens (p30, p54, p22, p10, p72, and CD2v) were performed under the same conditions. Briefly, ELISA plates (Costar Laboratories) were coated with 100 *µ*L of 1 *µ*g/mL ASFV recombinant proteins in 0.05 M bicarbonate/carbonate buffer (pH 9.6) and incubated overnight at 4°C. The plates were then washed three times with PBS containing 0.05% Tween 20 (PBST) and blocked with 200 *µ*L PBS containing 5% skim milk for 2 h at 37°C. After washing with PBST, the plates were incubated for 30 min at 37°C with 100 *µ*L of serum sample diluted 1 : 100 in PBS containing 1% skim milk. Following five washes with PBST, the plate was incubated with 100 *µ*L of horseradish peroxidase (HRP)-labelled goat anti-pig IgG antibody (Boster Laboratories) at a 1 : 30000 dilution for 30 min at 37°C. Finally, the plates were washed five times again and incubated with 100 *µ*L of substrate (0.1 mg/mL tetramethylbenzidine (TMB, Sigma), 0.1 M acetate buffer (pH 5.6), 1 mM urea hydrogen peroxide) in the dark for 10 min at 37°C. Colour development was halted by the addition of 50 *µ*L of 2 M H_2_SO_4_, and then *A*_450_ was read on a universal microplate reader (ELx800, Bio-Tek Instruments). *A*_450_ values of the serum samples collected before the challenge experiments were used as a negative control and indicated as N0 (also as 0 DPI). *A*_450_ values of serum samples at different DPIs are indicated as P. The dynamics of serum IgG responses to ASFV antigens were reflected by calculating the P/N0 values at different time points, and positive conversion was identified when the P/N0 value was ≥2.1.

### 2.6. Oral Fluid sIgA Responses to ASFV Antigens in Challenged Pigs

sIgA-specific indirect ELISAs were used to detect oral fluid sIgA responses to ASFV antigens (p30, p54, p22, p10, p72, and CD2v). The plates were coated and blocked as described for the serum IgG assays. The blocked plates were incubated with 100 *µ*L of oral liquid samples diluted 4 : 1 in PBS containing 5% skim milk for 2 h at 37°C. The plates were then washed five times and incubated with 100 *µ*L of HRP-labelled goat anti-pig IgA (BETHYL Laboratories) diluted 1 : 10000 for 1 h at 37°C. Finally, the plates were developed, and the absorbance was measured as described above. The dynamics of oral fluid sIgA responses to ASFV antigens were reflected by calculating the P/N0 values at different time points, and positive conversion was identified when the P/N0 value was ≥2.1, as described for serum IgG.

## 3. Results

### 3.1. Disease Signs and Viremia Detection in ASFV-Infected Pigs

**Figure 1:**
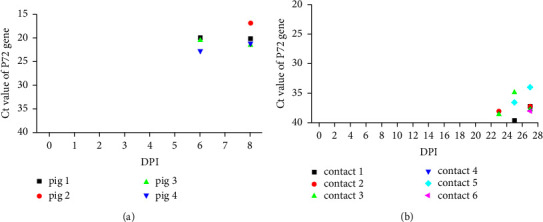
Viremia detection in serum samples from HLJ/18-infected pigs (a) and from SD/DY-I/21 or HLJ/HRB1/20-contact infected pigs (b). The differently shaped coloured dots represent individual pigs. Contact 1 and contact 2, contact 3 and contact 4, and contact 5 and contact 6 were cohoused with pigs that were intramuscularly inoculated with 10^3^ TCID_50_ SD/DY-I/21, 10^6^ TCID_50_ SD/DY-I/21, and 10^6^ TCID_50_ HLJ/HRB1/20 from the first day of challenge, respectively.

The inoculated pigs were observed daily for disease signs and death (Table 1). Pigs began to show disease signs, including anorexia and lethargy, high fever, and depression, from 4 DPI in the HLJ/18-infected group and finally died at approximately 8 DPI. However, the HLJ/18-7GD-infected group remained clinically healthy and survived the 60-day observation period. In the SD/DY-I/21 contact infected group, three pigs started to have fever at 4 DPI, 5 DPI, and 26 DPI and subsequently displayed clinical signs, including arthroncus and phyma, beginning at 17 DPI. No disease signs were observed in the last pig in this group, as well as the HLJ/HRB1/20 contact infected pigs. All of the pigs in contact with SD/DY-I/21 or HLJ/HRB1/20 survived for the duration of the 28-day observation period [8, 9]. The identity of the ASFV genome in serum by qPCR is shown in Figure 1. The results showed that the earliest detectable viral nucleic acid in serum was at 6 DPI in HLJ/18-infected pigs, while it was at 23 DPI and 25 DPI in naturally attenuated isolate SD/DY-I/21-infected and HLJ/HRB1/20-infected pigs, respectively. However, no ASFV genome was detected throughout the observation period in the HLJ/18-7GD-infected group.

### 3.2. Antibody Responses to ASFV Antigens in Field Virulent Isolate HLJ/18-Infected Pigs

**Figure 2:**
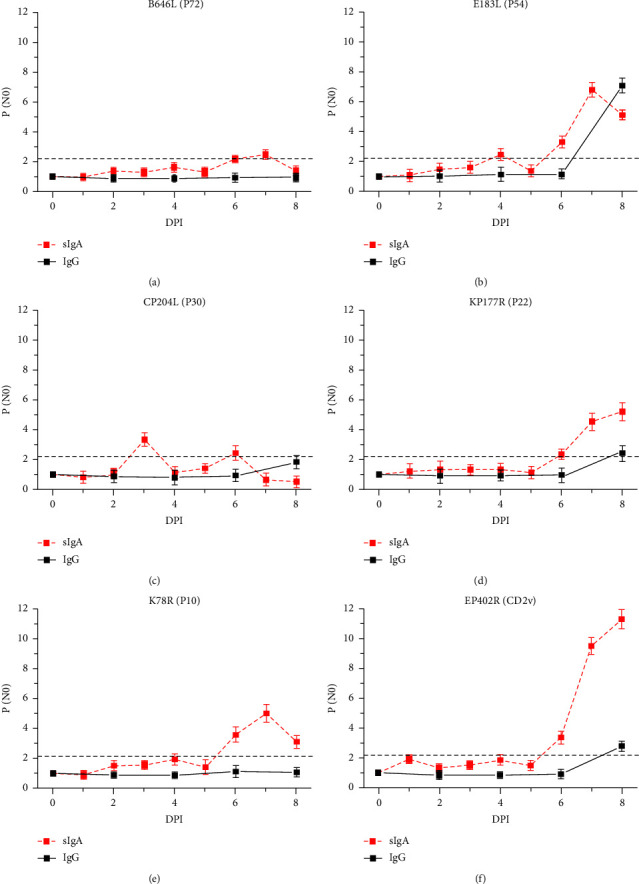
Serological IgG responses (indicated as black squares with a solid line) and mucosal sIgA responses (indicated as red squares with a dotted line) to ASFV recombinant proteins in the pigs orally and nasally inoculated with 10^5^ HAD_50_ of the virulent field genotype II isolate HLJ/18. ELISA plates coated with B646L/P72 (a), E183L/P54 (b), CP204L/P30 (c), KP177R/P22 (d), K78R/P10 (e), and EP402R/CD2v (f) proteins were used to determine specific antibody responses at different DPIs. The serological IgG responses were analysed using mixed serum samples from four pigs. Positive conversion was identified when the P/N0 value was ≥2.1 and is indicated by a grey dotted line. The data represent the mean and SD of three parallel experiments.

The quantitative antibody responses in serum and oral liquid samples from the pigs orally and nasally inoculated with 10^5^ HAD_50_ of field virulent genotype II isolate HLJ/18 are shown in Figure 2. The latest sIgA-positive conversion in oral liquid appeared at 6 DPI for the six antigens, while the earliest IgG-positive conversion in serum appeared at 8 DPI for only three antigens, indicating that the immune mucosal antibody sIgA response to ASFV infection occurred significantly earlier than the serological antibody IgG response. The first sIgA-positive conversion for P72 appeared at 6 DPI and then slightly dropped to a negative level (Figure 2(a)). The first sIgA-positive conversion for P54 appeared at 4 DPI. After a short period of negative transition, the P/N0 value for sIgA increased significantly again at 6 DPI and maintained a high positive level until 8 DPI (Figure 2(b)). The sIgA secretion regularity for P30 was similar to that for P54, but the earliest sIgA-positive conversion for P30 was at 3 DPI, with a subsequent second sIgA-positive conversion at 6 DPI without further increasing the P/N0 value (Figure 2(c)). The first sIgA-positive conversion for P22, P10, and CD2v appeared at 6 DPI, similar to that for P72, and this high positive level was maintained to 8 DPI, as it was for P54 (Figures 2(d)–2(f)). Moreover, among all the antigens, P10 showed the highest sIgA response at the first sIgA-positive conversion (Figure 2(e)), and CD2v showed the highest sIgA response throughout the whole detection period to 8 DPI (Figure 2(f)). Only P54, P22, and CD2v showed IgG-positive conversion at 8 DPI. A more significant increase in the P/N0 value of IgG was observed for P54 from the sixth day to the eighth day after inoculation compared to P22 and CD2v (Figures 2(b), 2(d), and 2(f)). P54 also showed the highest IgG response at 8 DPI, which was higher than the corresponding sIgA response (Figure 2(b)). Neither IgG-positive conversion nor sIgA-positive conversion occurred in the other two unchallenged control pigs.

### 3.3. Antibody Responses to ASFV Antigens in Artificially Attenuated Strain HLJ/18-7GD-Inoculated Pigs

**Figure 3:**
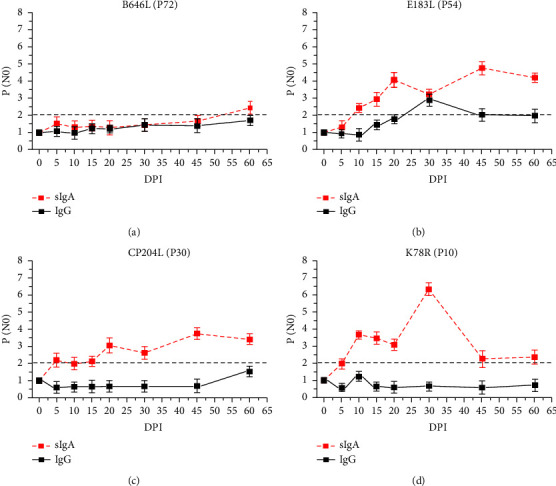
Serological IgG responses (indicated as black squares with a solid line) and mucosal sIgA responses (indicated as red squares with a dotted line) to ASFV recombinant proteins in pigs orally and nasally inoculated with 10^6^ TCID_50_ of the artificially attenuated strain HLJ/18-7GD. ELISA plates coated with B646L/P72 (a), E183L/P54 (b), CP204L/P30 (c), and K78R/P10 (d) proteins were used to determine specific antibody responses at various DPIs. The serological IgG responses were analysed using mixed serum samples from four pigs. Positive conversion was identified when the P/N0 value was ≥2.1 and is indicated by a grey dotted line. The data represent the mean and SD of three parallel experiments.

According to the analysis of antibody responses to ASFV antigens in the pigs orally and nasally inoculated with 10^6^ TCID_50_ of the artificially attenuated strain HLJ/18-7GD, the earliest positive conversion was found for P30, the highest sIgA response at the first sIgA-positive conversion was found for P10, the second-highest sIgA response throughout the whole detection period was found for P54, and a low sIgA level was maintained for P72; thus, these antigens were selected as the detection antigens for analysing the dynamics of antibody responses in artificially attenuated strain HLJ/18-7GD-inoculated pigs (as the CD2v gene is deficient in the artificially attenuated strain HLJ/18-7GD, CD2v, which showed the highest sIgA response, was not suitable as a detection antigen for this strain). As shown in Figure 3, except for P72, the sIgA levels of the other three antigens were all significantly higher than the IgG levels. Moreover, the IgG levels associated with the antigens remained negative during the whole detection period, except for P54, which showed a positive conversion at 30 DPI. The first sIgA-positive conversion for P72 appeared at 60 DPI, and that for P54 appeared at 10 DPI and remained higher than the sIgA-positive level throughout detection (Figures 3(a) and 3(b)). The earliest sIgA-positive conversion among the antigens was found for P30 at 5 DPI, and a weak positive level was maintained until 15 DPI. Then, a significant increase occurred again and remained higher than the sIgA-positive level until 60 DPI (Figure 3(c)). Among the selected antigens, the highest sIgA response at both the first sIgA-positive conversion at 10 DPI and throughout the detection period to 30 DPI was found for P10. After the first sIgA-positive conversion, P10 was associated with an sIgA-positive level until the last monitoring day at 60 DPI (Figure 3(d)).

## 4. Antibody Responses to ASFV Antigens in Contact Transmission-Infected Pigs

**Figure 4:**
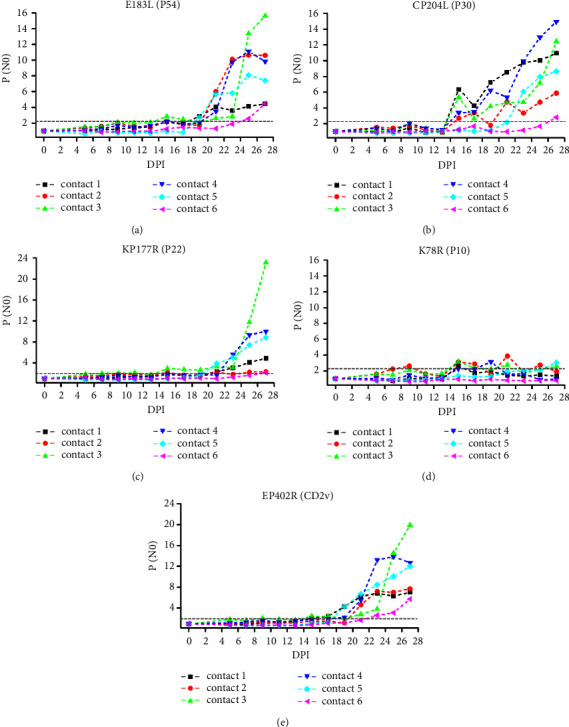
Serological IgG responses to ASFV recombinant proteins in contact transmission-infected pigs. ELISA plates coated with CP204L/P30 (a), E183L/P54 (b), K78R/P10 (c), KP177R/P22 (d), and EP402R/CD2v (e) proteins were used to determine specific antibody responses at various DPIs. Contact 1 and contact 2, contact 3 and contact 4, and contact 5 and contact 6 were cohoused with pigs that were intramuscularly inoculated with 10^6^ TCID_50_ of SD/DY-I/21, 10^3^ TCID_50_ of SD/DY-I/21, and 10^6^ TCID_50_ of HLJ/HRB1/20 from the first day of challenge, respectively. Positive conversion was identified when the P/N0 value was ≥2.1 and is indicated by a grey dotted line.

Low-virulence ASFVs, including naturally occurring genotype II variants and genotype I viruses, have recently emerged in domestic pigs in China and caused chronic infections [8, 9]. Experimental contact pigs cohoused with the infected pigs could truly reflect the natural infection of pigs in the field. Therefore, we analysed the antibody responses in four pigs cohoused with low-virulent genotype I SD/DY-I/21-inoculated pigs and two pigs cohoused with the attenuated genotype II HLJ/HRB1/20-inoculated pigs. As shown in Figure 4, an obvious IgG response appeared for all antigens except P10, and the earliest IgG response was exhibited for P30 at approximately 15 DPI. The IgG response to CD2v was higher in all six pigs than the responses to the other antigens. The IgG levels continued to rise when the IgG response occurred in all animals, and for the same antigen, most animals showed an IgG response on the same day. Furthermore, the IgG response kinetics in the contact transmission group were independent of whether cohoused pigs were intramuscularly inoculated with high or low doses (contact 1–4) and of whether cohoused pigs were inoculated with genotype I or genotype II attenuated strains (contact 3–6).

**Figure 5:**
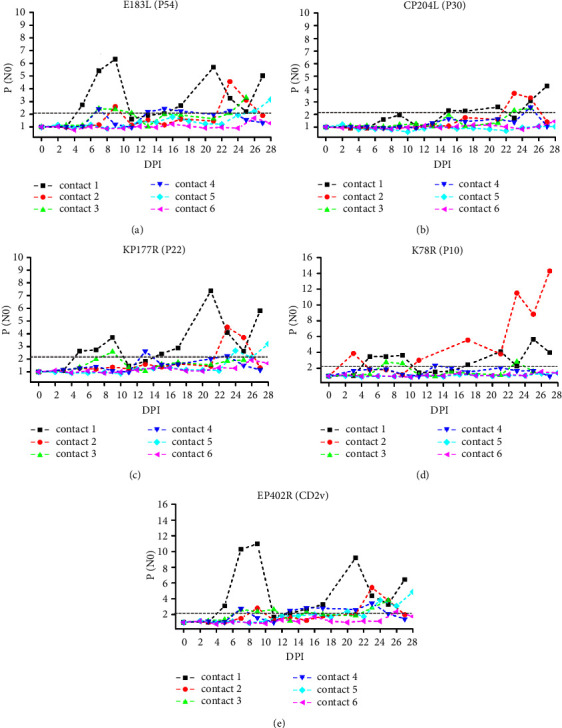
sIgA responses to ASFV recombinant proteins in contact transmission-infected pigs based on oral swab samples. ELISA plates coated with CP204L/P30 (a), E183L/P54 (b), K78R/P10 (c), KP177R/P22 (d), and EP402R/CD2v (e) proteins were used to determine specific antibody responses at various DPIs. Contact 1 and contact 2, contact 3 and contact 4, and contact 5 and contact 6 were cohoused with pigs that were intramuscularly inoculated with 10^6^ TCID_50_ of SD/DY-I/21, 10^3^ TCID_50_ of SD/DY-I/21, and 10^6^ TCID_50_ of HLJ/HRB1/20 from the first day of challenge, respectively. Positive conversion was identified when the P/N0 value was ≥2.1 and is indicated by a grey dotted line.

The sIgA responses in the six contact pigs were also analysed and are shown in Figure 5. To analyse the differences in antibody dynamics for individuals during contact transmission of naturally attenuated ASFV strains and avoid the collective effect of oral fluid on individual analysis, sIgA response analysis was performed using oral swabs. In contrast to the IgG response pattern, the corresponding sIgA level to the six antigens fluctuated greatly, which was perhaps because oral swabs are more difficult to standardize in sampling than serum. The corresponding sIgA level associated with the same antigen varied greatly among the six pigs, which was perhaps related to the early onset but short duration of mucosal immunity and the difference in infection time among the animals. As early as approximately 4–7 DPI, several animals showed sIgA responses. CD2v, P10, P54, and P22 were associated with strong sIgA responses, while P30 was associated with a moderate sIgA response. Notably, contacts 1 and 2, which showed relatively higher sIgA responses, were cohoused with pigs that were inoculated with high-dose SD/DY-I/21.

## 5. Discussion

The host immune response to ASFV is a very complex process involving various parts of cellular and humoral immunity [31–33]. Since oral and nasal transmission is the common transmission route of ASFV [17, 18], local mucosal immunity dominated by the mucosal barriers of the respiratory and digestive tracts is considered to play an important role in the host defence mechanisms against ASFV infection. Currently, prevention and control of ASF are extremely difficult due to the widespread prevalence of infections with naturally attenuated ASFV strains causing mild or no clinical signs in animals in endemic areas [8–10, 34]. Low levels of virus loading, intermittent shedding, and large individual differences characterize infections with naturally attenuated ASFV strains, making conventional nucleic acid detection difficult. Therefore, it is of great significance to analyse the dynamics of different antibody responses to the different antigens in attenuated ASFV-infected pigs for early diagnosis method establishment in the future. Here, a systematic analysis of oral fluid mucosal sIgA and serological IgG responses in HLJ/18-, HLJ/18-7GD-, SD/DY-I/21-, and HLJ/HRB1/20-infected pigs was performed.

The natural infective routes were simulated by infecting pigs with oral and nasal inoculations. Our results showed that the earliest detectable viral nucleic acids in serum were at 6 DPI in HLJ/18-infected pigs and at 23 DPI and 25 DPI in naturally attenuated isolate SD/DY-I/21-infected and HLJ/HRB1/20-infected pigs, respectively. Previous reports showed that the earliest detectable virus excretion in blood appeared at 3 DPI in highly virulent Georgia 2007/1-infected and Pol16/DP/OUT21-infected pigs [10, 35]. Here, we found that the sIgA responses to the selected ASFV antigens in the oral fluid samples were significantly stronger than the IgG responses in the serum samples in HLJ/18-infected (Figure 1) and HLJ/18-7GD-infected pigs (Figure 2), and the earliest sIgA-positive conversion in oral fluid occurred at 3 DPI in the field virulent HLJ/18-infected pigs. Therefore, we proposed that the mucosal immune response occurred earlier than the serum immune response to ASFV and even began at the same time the viral nucleic acid was detected.

By comparing the difference in antibody response kinetics between virulent and attenuated ASFV strains, we found that mucosal sIgA responses occurred earlier and were stronger than serological IgG responses after virulent isolate HLJ/18 infection (Figure 2). The secretion of oral mucosal sIgA increased significantly after artificially attenuated HLJ/18-7GD infection, while the serological IgG levels were almost always negative throughout the monitoring process (Figure 3). These results suggested that common serological IgG tests may have missed the detection of attenuated ASFV-infected animals. The serological IgG response against specific antigens was significantly increased in both genotype I (SD/DY-I/21) and genotype II (HLJ/HRB1/20) naturally attenuated strains and was independent of the dose of cohoused inoculated animals. The oral swab sIgA response showed significant individual differences from the serological IgG response in the contact groups, which may be related to the difference in the dose of ASFV infection among cohoused pigs. On the contrary, owing to the poor uniformity of oral samples, and the influence of behaviours on the content of oral samples, including drinking water and eating food, oral samples are more difficult to standardize in collection than serum samples. However, earlier antibody level elevations appeared for oral swab sIgA than serum IgG (Figures 4 and 5). Together, these results indicated that mucosal immunity plays an important role in the early diagnosis of ASFV infection, especially infection with low-virulence ASFV strains without significant clinical signs and difficult detection of viral excretion.

The identification of ASFV immunodeterminants eliciting a strong and sustained antibody response is a step towards the development of more sensitive and specific diagnostic tools. Here, P30 showed the earliest sIgA-positive conversion both in oral fluid from HLJ/18- and HLJ/18-7GD-challenged pigs (Tables 2 and 3), indicating that P30 is a promising target for ASFV diagnosis with expected earlier detection and higher sensitivity. P54 showed earlier sIgA responses and stronger serological IgG responses in HLJ/18- and HLJ/18-7GD-challenged pigs (Tables 2 and 3) and in contact transmission-infected pigs. However, due to the variation in the amino acid sequence of P54, the ASFV antibody detection accuracy was low in West Africa [36]; thus, whether it is suitable as a target antigen for ASFV diagnosis requires further investigation. P72 is often used as the target antigen for ASFV genotyping and serological diagnosis due to its high sequence conservation [37–39]. However, we found that pigs challenged with HLJ/18 or HLJ/18-7GD or infected by contact transmission retained low serological and mucosal antibody levels against P72 (Figures 2 and 3), suggesting that P72 may be deficient in sensitivity as an immunological diagnosis target of ASFV. CD2v was associated with a significant increase in sIgA P/N0 values in the first sIgA-positive conversion and continued to increase to the strongest sIgA response in HLJ/18-infected pigs (Figure 2 and Table 2) and showed strong sIgA responses in contact transmission-infected pigs. Given the deletion or modification of the CD2v gene in the ASFV candidate vaccine currently reported [40, 41], CD2v might be an ideal target for the sensitive diagnosis of naturally attenuated ASFV-infected pigs and for differential diagnosis after the application of the gene deletion vaccine. In contrast to the HLJ/18-inoculated group, both HLJ/18-7GD-inoculated pigs and contact transmission-infected pigs showed the highest P/N0 value against P10 (Table 3). These results indicate that P10 is promising as a diagnostic target for sIgA antibody detection in attenuated ASFV-infected pigs, which remained clinically healthy, survived and were negative for ASFV genome detection. However, because the variety and number of pigs involved in this study are limited, more samples from different animals should be collected for further verification of the above inference, although we improve the reliability and reference of experimental data by ensuring the quality of animals and the environmental conditions of breeding. In addition, the significantly earlier secretion of mucosal sIgA in oral samples than serum IgG is conducive to the early screening of ASFV. As a noninvasive method, sIgA detection targets the oral fluid of animals, which does not easily to cause animal stress and avoids the risk of cross-infection caused by blood collection. However, in the future establishment of an ASFV early diagnosis method based on the mucosal response level, attention should be given to standardized collection studies of mucosal samples such as oral fluid.

In this study, using established ELISA methods, the dynamics of serological and mucosal antibodies against 6 ASFV antigens after direct or indirect infection with four different virulent ASFV strains were determined. Compared with pigs infected with the virulent HLJ/18 strain, those infected with either artificially (HLJ/18-7GD) or naturally attenuated (SD/DY-I/21 and HLJ/HRB1/20) strains survived showing no and chronic disease signs in the HLJ/18-7GD-infected group and in naturally attenuated strains infected groups, respectively. However, oral fluid sIgA responses preceded serological IgG in all infection groups regardless of virulence and infection routes (direct or contact). Moreover, the differential analysis of antibody responses to different ASFV antigens provides us with references for screening target antigens and antibodies for the early diagnosis of ASFV infection. For example, P54 can be used as a dominant target for serological IgG diagnosis, while P30, CD2v, P54, P22, and P10 can be used for mucosal sIgA diagnosis.

## Figures and Tables

**Table 1:** Disease signs of pigs inoculated with different doses of ASFV isolates.

ASFV virus	Dose	Pig no.	The earliest time when disease signs appeared (days post-inoculation)					
			Fever	Arthroncus	Rhyma	Limp	Depression	Death
HLJ/18	10^5^HAD_50_	1	4	—^a)^	—	—	5	7
		2	4	—	—	—	5	8
		3	4	—	—	—	5	8
		4	5	—	—	—	5	8

HLJ/18-7GD (non-HAD)	10^6^TCID_50_	1	—	—	—	—	—	NA^b)^
		2	—	—	—	—	—	NA
		3	—	—	—	—	—	NA
		4	—	—	—	—	—	NA

SD/DY-I/21 (non-HAD)^c)^	Contact	1	—	—	—	—	—	NA
		2	26	17	—	—	—	NA
		3	5	—	25	—	—	NA
		4	4	—	—	—	—	NA

HLJ/HRB1/20 (non-HAD)^c)^	Contact	5	—	—	—	—	—	NA
		6	—	—	—	—	—	NA

^a)^No disease sign observed. ^b)^The pig survived from the infection. ^c)^Diseases signs of the contact infected pigs referred to our previous reports [8, 9].

**Table 2:** Summary of the key time points of the sIgA response to ASFV recombinant proteins in field virulent isolate HLJ/18-infected pigs.

ASFV recombinant proteins	The first sIgA-positiveconversion^*∗*^ time	The max P/N0 value	The time of the max P/N0 value
P72	6 DPI	2.486	7 DPI
P54	4 DPI	6.831	7 DPI
P30	3 DPI	3.333	3 DPI
P22	6 DPI	5.215	8 DPI
P10	6 DPI	5.01	7 DPI
CD2v	6 DPI	11.32	8 DPI

^*∗*^Positive conversion was identified when P/N0 value ≥ 2.1.

**Table 3:** Summary of the key time points of the sIgA response to ASFV recombinant proteins in artificially attenuated strain HLJ/18-7GD-inoculated pigs.

ASFV recombinant proteins	The first sIgA-positiveconversion^*∗*^ time	The max P/N0 value	The time of the max P/N0 value
P72	60 DPI	2.444	60 DPI
P54	10 DPI	4.742	45 DPI
P30	5 DPI	3.755	45 DPI
P10	10 DPI	6.356	30 DPI

^*∗*^Positive conversion was identified when P/N0 value ≥2.1.

## Data Availability

The data that support the findings of this study are available from the corresponding author prof. Z.F., upon reasonable request.

## Supplementary Materials

Supplementary Materials Identification of the purified ASFV proteins CD2v, p10, p22, p54, p30, and p72 using 12% SDS-polyacrylamide gel electrophoresis with coomassie blue stained (Figure S1).
