# Supplementary material for: Dynamics of Serological and Mucosal Antibody Responses against African Swine Fever Viruses in Experimentally Infected Pigs
Source: Transbound Emerg Dis. 2023 Feb 25;2023:9959847. doi: 10.1155/2023/9959847 (PMC12017000; doi:10.1155/2023/9959847)
Supplement: Supplementary Materials — Identification of the purified ASFV proteins CD2v, p10, p22, p54, p30, and p72 using 12% SDS-polyacrylamide gel electrophoresis with coomassie blue stained (Figure S1). [file 9959847.f1.docx]

**Supplementary Material**

The recombinant plasmids pET28a-p72, pET32a-p10, pET21a-p22, pET21a-p54 for prokaryotic expression, and pFastBac-sumo-p30, pFastBac-CD2v for baculovirus expression system were designed and constructed according to the complete genome of ASFV isolate Pig/HLJ/2018 (Genbank: MK333180.1). The predicted molecular weights of the recombinant proteins were approximately 42 Kda (P72, 1-325aa and contains 6 Kda tags carried by plasmid pET28a), 29 Kda (P10, contains 19 Kda tags carried by plasmid pET32a), 21 Kda (P22), 21 Kda (P54), 45 Kda (P30, contains 20 Kda tags carried by plasmid pFastBac-sumo), 20 Kda (CD2V, 21-206aa). The recombinant proteins were purified using Ni NTA affinity columns (Genscript Laboratories), and identified by SDS-polyacrylamide gel electrophoresis (Figure S1).


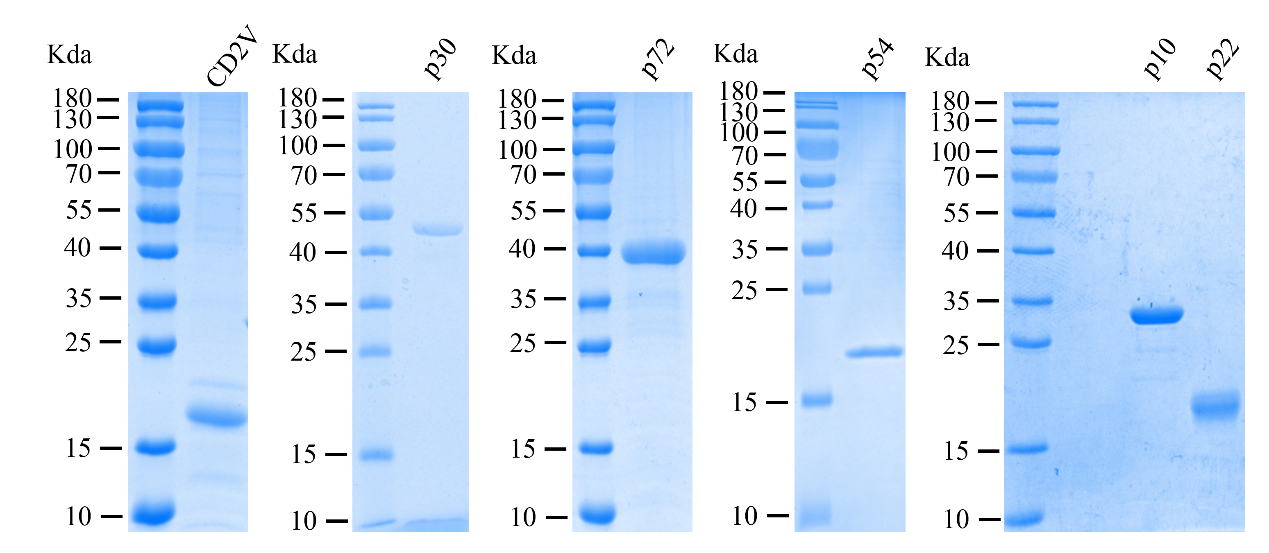


Figure S1. Identification of the purified ASFV proteins CD2v, p10, p22, p54, p30 and p72 using 12% SDS-polyacrylamide gel electrophoresis with coomassie blue stained.
